# Screening blood-fed mosquitoes for the diagnosis of filarioid helminths and avian malaria

**DOI:** 10.1186/s13071-015-0637-4

**Published:** 2015-01-13

**Authors:** Carina Zittra, Zsanett Kocziha, Szilárd Pinnyei, Josef Harl, Katrin Kieser, Alice Laciny, Barbara Eigner, Katja Silbermayr, Georg G Duscher, Éva Fok, Hans-Peter Fuehrer

**Affiliations:** Institute of Parasitology, Department of Pathobiology, University of Veterinary Medicine, Vienna, Austria; Department of Parasitology and Zoology, Faculty of Veterinary Science, Szent István University, Budapest, Hungary; Institute of Animal Sciences and Wildlife Management, Faculty of Agriculture, Szeged University, Hódmezővásárhely, Hungary

**Keywords:** Culicidae, *Dirofilaria*, *Setaria tundra*, Blood-fed, Avian malaria, *Cytochrome c oxidase I*, *Cytochrome b*, Hungary

## Abstract

**Background:**

Both *Dirofilaria repens* and recently *D. immitis* are known to be endemic in Hungary. As one of several recent cases, the fatal case of a dog infested with *D. immitis* in Szeged, Southern Hungary*,* received attention from the media. Hence it was decided to catch mosquitoes in the garden where the dog lived to screen for filarioid helminths and *Plasmodium* spp. using molecular tools.

**Methods:**

Mosquitoes were caught in Szeged, in the garden where the infected dog was kept, in July 2013 with M-360 electric mosquito traps and were stored in ethanol until further procedure. Female mosquitoes were classified to genus level by morphology. Each mosquito was homogenized and analyzed for filarioid helminths and avian malaria using standardized PCR techniques. Positive mosquito samples were further identified to species level by comparing a section of the mitochondrial *COI* gene to GenBank**®** entries.

**Results:**

In this study, 267 blood-fed mosquitoes were caught in July 2013 in Szeged. Subsequent molecular screening revealed that not only *D. immitis* was present in the analyzed specimens but also DNA of *D. repens*, *Setaria tundra* and *Plasmodium* spp. was confirmed.

**Conclusions:**

The analysis of blood-fed mosquitoes for the diagnosis of *Dirofilaria* spp. and other mosquito-borne pathogens seems to be an adequate technique to evaluate if filarioid helminths are present in a certain area. Usually only unfed female mosquitoes are analyzed for epidemiological studies. However, blood-fed mosquitoes can only be used for screening if a pathogen is present because the role of the mosquito as vector cannot be classified (blood of bitten host). Furthermore, *Setaria tundra* was confirmed for the first time in Hungary.

## Background

The city of Szeged is situated in the Southern Great Hungarian Plain, near the mouth of the River Maros and the lower reaches of the River Tisza. The natural mosquito density in this area is mainly influenced by prevailing rainfall and the flood of the River Tisza, which has two maxima annually, occurring in April after snowmelt and at the end of November. As a wetland, this region is known to provide many breeding habitats for mosquitoes (Diptera: Culicidae) [1]. Species belonging to the genera *Culex*, *Culiseta, Aedes*, *Anopheles*, and *Coquillettidia* have been mentioned as being incriminated in the transmission cycle of filarioidal infections [2].

Both *D. repens* and *D. immitis* are emerging causative agents of parasitic zoonoses in Europe occurring in domestic and wild carnivores and are transmitted by mosquitoes [3]. Mostly climatic changes are considered to be the main reason for the expansion of geographic ranges in parasites [3] and also influence the development and longevity of their hosts; both the hosts and parasites are currently expanding to northern areas in Europe. These findings were underlined by an increasing number of diagnosed filarioid infections in countries such as Austria, Germany, the United Kingdom, the Netherlands, Sweden, and Hungary [2]. Further global drivers like trade, travel (movement of dogs across Europe), and insecticide resistance are also mentioned as being responsible for a higher risk of animal and human infections [3].

In Hungary imported dogs infected with *D. immitis*, the most frequent causative agent of canine and feline cardiopulmonary filarioidosis, were already reported in 1982 [4]. However, the first autochthonous case of a *D. immitis* infection in a dog, originating from Jász-Nagykun-Szolnok, was reported in 2007 [5]. Since that time, Hungary is considered to be a heartworm endemic country and autochthonous filarioid infections caused by *D. immitis* have since been examined in Hungarian red foxes (*Vulpes vulpes*) and golden jackals (*Canis aureus*) [6]. In contrast, cases of filarioid infections caused by *D. repens*, the causative agent of canine and feline subcutaneous and ocular filarioidosis, were not reported until 1997 [7]. It was not clear that the latter infection was really acquired in Hungary [8], so the first autochthonous cases of *D. repens* infection were reported in 1998 and 1999 [9,10].

The primarily boreal filarioid parasite *Setaria tundra* is the causative agent of setariosis and can act as a significant pathogen in domestic reindeer (*Rangifer tarandus*), but also in roe deer (*Capreolus capreolus*), moose (*Alces alces*), and other cervids [11,12]. *S. tundra* is known to be endemic in Central Europe (e.g. Germany and Austria), with roe deer as the main vertebrate host [13]. In Hungary, only *S. equina* has been reported in horses (*Equus ferus caballus*) [14] and *S. cervi* in red deer (*Cervus elaphus*) [15]. However, *S. tundra* had never been reported from Hungary until now. Main vectors are mosquitoes belonging to the genera *Aedes, Anopheles* and *Ochlerotatus* [13], of which *Aedes* spp. are considered the most important and competent vectors. In Finland, *S. tundra* caused severe outbreaks of peritonitis, with significant economic losses in semi-domestic reindeer in 1973 and 2003–2005 [16]. However, human infections have not been described so far.

In principle, the known main vectors of *D. immits*, *D. repens*, and *S. tundra* are mosquito species belonging to the genera *Aedes*, *Anopheles, Ochlerotatus*, and *Culex* [16,13]. Mosquitoes are distributed worldwide with over 3,500 species. Fifty mosquito species belonging to eight genera (*Anopheles*, *Aedes*, *Ochlerotatus*, *Coquillettidia*, *Culex*, *Culiseta*, *Orthopodomyia*, and *Uranotaenia*) have been documented in Hungary to date [17]. The presence of 17 mosquito species in Szeged, belonging to the genera *Culex, Aedes, Ochlerotatus, Culiseta*, and *Anopheles,* was reported in 2006 [1] and underlines the importance of fast and efficient screening methods in order to gain an overview of the present infestation rate and further spread of filarioid helminths in this country.

As one of several recent cases, the fatal case of a dog infested with *D. immitis* in Szeged, Southern Hungary*,* received attention from the media and served as the initial reason to perform this study. The main aim of this study was the examination of the usefulness of screening blood-fed mosquitoes for the presence of filarioid helminths and avian malaria.

## Methods

Mosquitoes were caught in July 2013 with an M-360 electric mosquito trap in Szeged, Southern Hungary. They were stored in 75% ethanol until further processing and were transported to the Institute of Parasitology at the University of Veterinary Medicine, Vienna. Due to the sampling method, female mosquitoes were only classified to the genus level by morphological standard characteristics using the keys by Schaffner *et al.* [18] and Becker *et al*. [19]. Treating each mosquito individually and not merging them into pools, is obligatory to define an accurate estimation of the real number of mosquitoes positive for filarioid helminths and/or avian malaria. Therefore, a 3-mm Tungsten Carbide Bead (Qiagen, Hilden, Germany) was added to each mosquito. After homogenization in a TyssueLyser II (Qiagen, Hilden, Germany), DNA was extracted using the DNeasy®blood and tissue isolation kit according to the manufacturer’s protocol. Afterwards the mosquitoes were analyzed for filarioid helminths by a conventional polymerase chain reaction (PCR), targeting an approximately 667 bp fragment of the filarioid mitochondrial *cytochrome oxidase subunit I* gene *(COI)* as reported previously [13,20,21].

In order to examine the presence of avian malaria, a nested PCR, targeting an approximately 500 bp fragment of the mitochondrial *cytochrome b* gene (*cytb*), was used [22,23].

Furthermore, PCR products positive for filarioid helminths or avian malaria were excised from the gel using the QIAquick® Gel Extraction Kit (Qiagen, Hilden, Germany) or were purified using Illustra™ ExoStar™ 1-Step, an enzymatic PCR and Sequencing Clean-up (GE Healthcare, Buckinghamshire, United Kingdom), for subsequent DNA sequencing. Afterwards consensus sequences were compared to GenBank® entries using BLAST (http://blast.ncbi.nlm.nih.gov). Mosquitoes that were positive for filarioid helminths and avian malaria were further specified to species level by barcoding (*COI* gene) as described previously [24].

In order to demonstrate the high genetic diversity of avian *Plasmodium* found at the Hungarian investigation site, a phylogenetic tree was calculated with avian malaria sequences retrieved from the NCBI database (http://www.ncbi.nlm.nih.gov/). A BLAST-search limited to the genus *Plasmodium* (taxid:5820) was performed against the NCBI nucleotide database with one of the *cytb* sequences obtained in the present study. The search resulted in a total of 3,824 BLAST hits. In order to obtain only those fragments overlapping with the *cytb* fragment analyzed, the BLAST hits were downloaded as aligned sequences in FASTA format. The GenBank® files were downloaded in addition to extract specific metadata (organism, country and host). The two files were then merged in Microsoft Excel, and all 650 BLAST hits with less than 400 bp were excluded from the dataset, resulting in a total of 3,175 sequences. The information on the host species was either retrieved from the GenBank® files or from the original publication, respectively; a total of 988 sequences originated from blood samples of bird hosts, 65 of which were excluded from the alignment due to the presence of ambiguous characters and/or stop codons. Including the 16 Hungarian samples, the alignment contained a total of 939 sequences. The sequences were aligned with Mafft v.7 [25], using the setting G-INS-i for accurate alignments. Using the program DAMBE v.5.2.78 [26], sequences were collapsed to 419 unique haplotypes, which were subsequently used for the phylogenetic tree calculation. A search for the best fitting substitution model was performed with JModeltest v.2.1.5 [27]. Based on the Akaike Information Criterion (AIC), TN93 + G was the best model. Finally, a Neighbor-Joining tree was calculated with 500 bootstrap replicates using MEGA v.6.06 [28].

## Results

In this study 267 blood-fed mosquitoes were caught with the M-360 electronic mosquito trap in Szeged, Southern Hungary in June 2013. The molecular screening revealed that not only *D. immitis* was present in the analyzed specimens. DNA of *D. repens, S. tundra*, and *Plasmodium* spp. was also confirmed. In total, 22 mosquitoes (8.2% of the total catch) were positive; four *Cx. pipiens* (Linnaeus 1785), one *Cx. modestus* (Ficalbi 1889), and one *Oc. caspius* (Pallas 1771) were positive for *D. immitis. D. repens* was detected in one *Oc. caspius* (Table 1). The finding in a single *Ae. vexans* (Meigen 1830) infected with *S. tundra* represents the first record of DNA of this parasite in Hungary. Additionally, avian malaria was detected in 16 specimens of *Cx. pipiens* (Table 2).

**Table 1:** **Filarioid helminths detected in blood-fed mosquitoes**

**Sample**	**Intermediate host/Mosquito**	**GenBank® entry**	**Pathogen**	**GenBank® entry**
HU11	*Culex (Culex) pipiens*	KM452929	*Dirofilaria immitis*	KM452920
HU73	*Ochlerotatus (Ochlerotatus) caspius*	KM452934	*Dirofilaria immitis*	KM452921
HU91	*Aedes (Aedimorphus) vexans*	KM452935	*Setaria tundra*	KM452922
HU151	*Culex (Culex) pipiens*	KM452937	*Dirofilaria immitis*	KM452923
HU201	*Culex (Culex) pipiens*	KM452943	*Dirofilaria immitis*	KM452924
HU217	*Culex (Culex) pipiens*	KM452945	*Dirofilaria immitis*	KM452925
HU236	*Culex (Barraudius) modestus*	KM452947	*Dirofilaria immitis*	KM452926
HU250	*Ochlerotatus (Ochlerotatus) caspius*	KM452948	*Dirofilaria repens*	KM452927

**Table 2:** **Mosquitoes infested with** ***Plasmodium spp.*** **(cytb gene)**

**Sample**	**Host/Mosquito**	**GenBank entry**	**Pathogen**	**GenBank entry**
HU5A	*Culex (Culex) pipiens*	KM452928	*Plasmodium* sp.	KM396866
HU5B	*Culex (Culex) pipiens*	KM452928	*Plasmodium* sp.	KM396867
HU29	*Culex (Culex) pipiens*	KM452930	*Plasmodium* sp.	KM396868
HU36	*Culex (Culex) pipiens*	KM452931	*Plasmodium* sp.	KM396869
HU42	*Culex (Culex) pipiens*	KM452932	*Plasmodium* sp.	KM396870
HU63	*Culex (Culex) pipiens*	KM452933	*Plasmodium* sp.	KM396871
HU128	*Culex (Culex) pipiens*	KM452936	*Plasmodium* sp.	KM396872
HU157	*Culex (Culex) pipiens*	KM452938	*Plasmodium* sp.	KM396873
HU172	*Culex (Culex) pipiens*	KM452939	*Plasmodium* sp.	KM396874
HU184	*Culex (Culex) pipiens*	KM452940	*Plasmodium* sp.	KM396875
HU185	*Culex (Culex) pipiens*	KM452941	*Plasmodium* sp.	KM396876
HU196	*Culex (Culex) pipiens*	KM452942	*Plasmodium* sp.	KM396877
HU210	*Culex (Culex) pipiens*	KM452944	*Plasmodium* sp.	KM396878
HU220A	*Culex (Culex) pipiens*	KM452946	*Plasmodium* sp.	KM396879
HU220B	*Culex (Culex) pipiens*	KM452946	*Plasmodium* sp.	KM396880
HU252	*Culex (Culex) pipiens*	KM452949	*Plasmodium* sp.	KM396881

**Figure 1:**
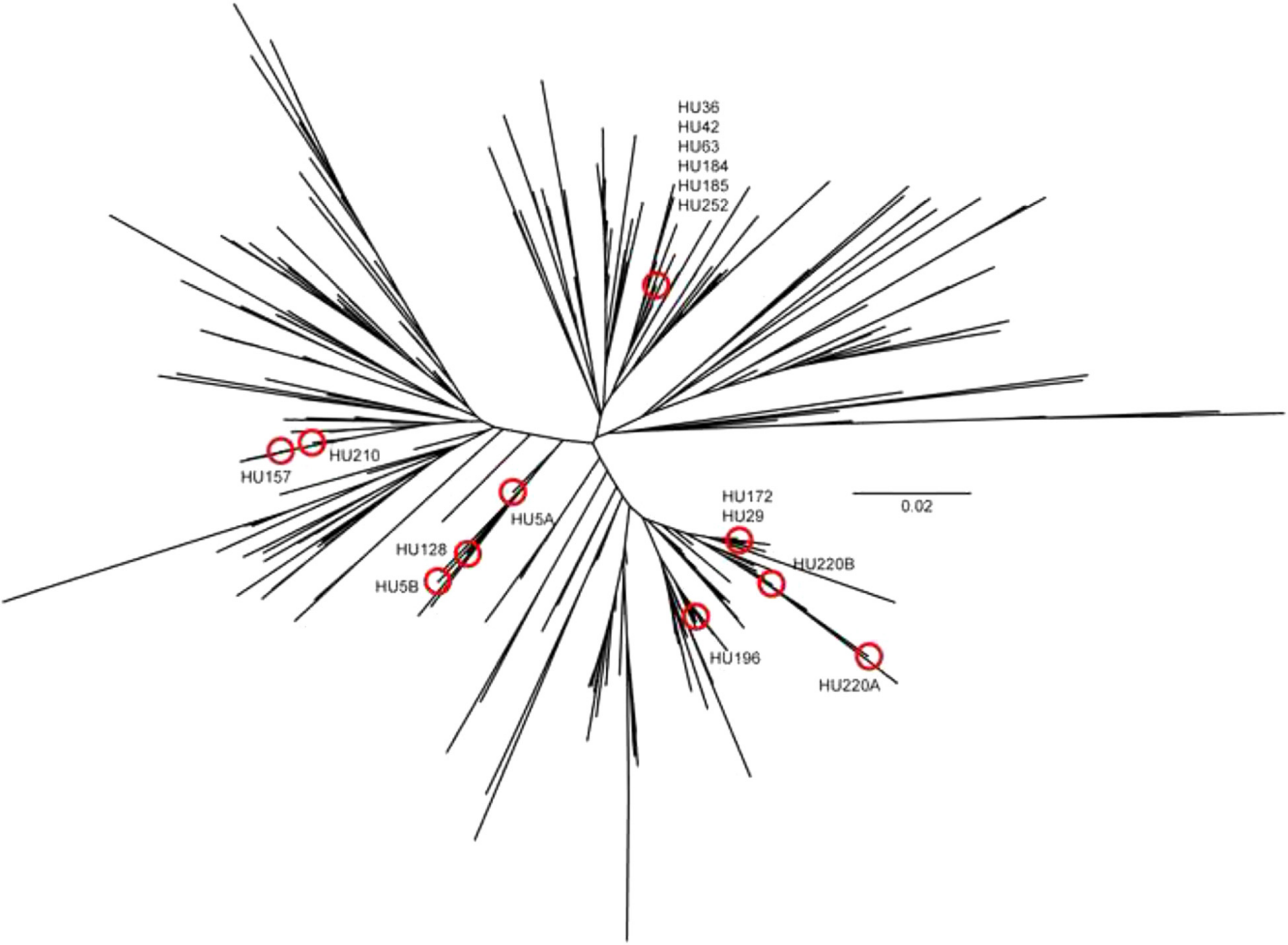
**Unrooted radial neighbour-joining tree.** *Plasmodium cytb* sequences of the present study and worldwide avian malaria sequences. Red circles mark tips of Hungarian Plasmodium lineages.

A Neighbor-Joining tree was calculated with 923 avian malaria *cytb* sequences from the NCBI database and the 16 *Plasmodium* sequences of the present study. The aim was to show the high genetic diversity of *Plasmodium* samples analyzed in the present study. The tree is displayed as an unrooted radial tree (Figure 1). Due to the high number of sequences and the short sequence length (400 bp), support values were low for most nodes and are not displayed in the tree. The 11 Hungarian *Plasmodium* haplotypes (11 haplotypes) cluster in four of the main clades in the tree. Other sequences in the respective clades originate from bird blood samples collected worldwide. The data show none of the Hungarian *Plasmodium* variants is unique to Europe, but that all haplotypes, respectively slightly deviating ones, are also present in other continents.

## Discussion

The analysis of blood-fed mosquitoes for the diagnosis of *Dirofilaria* spp. and other mosquito-borne pathogens seems to be an adequate technique to evaluate if parasites such as filarioid helminths are present in a certain area. Usually only unfed female mosquitoes are analyzed for epidemiological studies, because attracting female blood-fed mosquitoes is still problematic. Most sampling methods are based on the usage of carbon dioxide or lures imitating the human skin and similar attractants. These methods attract mainly unfed female mosquitoes searching for an adequate potential host. In contrast, blood-fed females seek resting places or are attracted by potential breeding sites. In this case the presence of a rain barrel, situated in the garden of the infected dog, might be the main reason for the high number of sampled blood-fed females.

Another important issue to be considered is that blood-fed mosquitoes can only be used for screening if a pathogen is present, whereas the role of the mosquito as a vector cannot be classified. The possibility of screening the blood of the bitten host is given by this method, because female mosquitoes take up microfilaria by feeding on infected animals, but the further development into the infecting stage L3 strongly depends on the vector efficiency and competence of the intermediate host, the mosquito [29]. Furthermore the development of the microfilaria within the mosquito itself is temperature-dependent, requiring about two weeks at a temperature of ≥ 26°C [29]. The longevity, seasonal appearance, and distribution of the individual mosquito depend on the mosquito species itself and on the prevailing climate [30].

The abundance of *D. repens* infections has increased since the first detection of *D. repens* in Hungary, especially in wet areas along the Danube and the River Tisza [31]. Though the mosquito collections can differ between natural and urban areas, Szeged, as a city, provides many potential breeding habitats like artificial and natural lakes, rain barrels and similar artificial water containers near houses, and rubbish dumps, which can easily be found by species belonging to the genus *Culex*. In contrast to *Aedes* and *Ochlerotatus,* numbers of *Culex* individuals can be considerably high even in seasons with a low amount of precipitation [1]. A previous study of mosquito collections observed *Culex pipiens molestus* and *Culex modestus* as the most prevalent mosquito species in Szeged [1]. In our study, 86.4% of mosquitoes infected with filarioid helminths and avian malaria belonged to the genus *Culex* (Tables 1 and 2). Thus, the vector competence of *Culex* species and the possibility to breed several generations per year, independent of precipitation and floods of the River Tisza, should be monitored.

## Conclusions

The molecular screening of blood-fed mosquitoes for filarioid helminths is an adequate tool to confirm the presence of *Dirofilaria* spp. and *Plasmodium* spp. This screening method supports a fast diagnosis of *Dirofilaria* and other pathogens and is necessary considering that climate change and globalization increase the risk of zoonotic *D. immitis* and *D. repens* in Central and Northern Europe [32]. Furthermore, DNA of *S. tundra* was confirmed for the first time in Hungary. Suitable studies on the distribution of *S. tundra* in Central Europe remain scarce and the possible health impact on the endemic cervid fauna in Hungary needs further epidemiological investigation.

## Abbreviations

COI: Cytochrome c oxidase subunit I
Cytb: Cytochrome b
